# Exploring the facilitators and barriers to achieving universal health coverage in Uganda: a qualitative study of the free healthcare policy

**DOI:** 10.1186/s12961-025-01334-8

**Published:** 2025-05-19

**Authors:** Prossy Kiddu Namyalo, Cyndirela Chadambuka, Lisa Forman, Beverley M. Essue, Freddie Ssengooba

**Affiliations:** 1 Institute of Health Policy, Management & Evaluation, University of Toronto, 155 College St, Suite 425, Toronto, ON M5T 3M6 Canada; 2 Dalla Lana School of Public Health, University of Toronto, 155 College St, Toronto, ON M5T 3M6 Canada; 3 School of Public Health, College of Health Sciences, Makerere University, P.O. Box 7072, Kampala, Uganda

## Abstract

**Background:**

Critical variations often occur between a state’s initial public policy goals and its implementation outcomes. After two decades, the implementation of the free healthcare policy in Uganda has not achieved the desired outcomes, and there is a lack of comprehensive contextual analysis applying implementation science approaches in the identification of barriers and facilitators. This study explores barriers and facilitators to the implementation of the free healthcare policy, drawing on the retrospective experiences of policymakers, policy advocates, policy supporters or influencers, policy implementers, and policy beneficiaries.

**Methods:**

We employed an exploratory qualitative study design and conducted 27 semi-structured interviews with key informants and 16 focus groups with users. Perspectives on implementation over time were collected by incorporating questions relating to the policy implementation journey from inception to 2023. The Consolidated Framework for Implementation Research guided data analysis to categorize and examine the barriers and facilitators to implementation. Two coders independently coded the data, which were thematically analysed with NVivo.14.

**Results:**

A total of five main factors were identified, synthesized, and categorized as barriers and facilitators with overlaps, namely: (i) financial resources, (ii) medicines and supply system, (iii) health human resources, (iv) infrastructure and functionality, and (v) equity and the FHP Implementation.

**Conclusions:**

Findings illustrate that policy implementation gaps are due to limited resources, political will that does not translate into sufficient allocation of funds, and incremental policy shifts that are not driving meaningful improvement in the health system. The findings explain why the free healthcare policy implementation has been unsuccessful and highlight the importance of investing in resources to support meaningful progress towards universal health coverage.

**Supplementary Information:**

The online version contains supplementary material available at 10.1186/s12961-025-01334-8.

## Introduction

Critical variations often occur between the initial public policy goals and the implementation outcomes. Policies are not always implemented as envisioned and do not necessarily achieve their intended results. Moreover, it is difficult to say which conditions or factors enable successful policy implementation because so much depends on the social, political and economic context and dynamics thereof [1–3]. Even where policy implementation appears successful, there is no guarantee that success will last [3, 4]. For instance, although user fees were anticipated to be beneficial in most sub-Saharan countries such as Uganda, they hindered the poor from accessing healthcare services and were costly to administer [5]; eight years after their introduction, user fees were abolished in Uganda, and the government introduced the Free Healthcare Policy (FHP) as a pathway to universal health coverage (UHC) [6–11].

UHC, which is included as part of Sustainable Development Goal (SDG) 3 on good health and wellbeing (specifically Target 3.8), aims to ensure all people receive the necessary healthcare services without facing financial hardship. On the basis of this definition, UHC aims to achieve equity access in service use and financial protection [8]. Free healthcare policies are key to the achievement of UHC by addressing financial barriers by eliminating formal fees at the point of service, constituting a pro-poor policy lever [9–11].

Ten days before the 2001 presidential election, in response to public concerns about high user fees hindering the utilization of healthcare services, President Yoweri Museveni (the incumbent) made an election promise that guaranteed free healthcare services for the whole population. After winning the election, policy implementation focused on rolling out a free healthcare policy [7, 10, 11]. However, despite the government providing free healthcare services for more than two decades, out-of-pocket (OOP) payments are still high. For example, in 2024, OOP payments were 79% of total health spending [12]. It is evident that if high OOP spending is required, it may limit people’s ability to access health services, resulting in unmet needs [13, 14] and catastrophic health expenditures and impoverishment [14–18]. Hence, after 24 years of implementation, the free health policy has yet to meaningfully achieve the desired outcome of financial protection for all people.

Policies do not fail or succeed on their own [5], thus warranting an understanding of the factors facilitating and hindering FHP implementation. Evidence regarding enabling and hindering factors for policy implementation is well documented [5, 19–21], although they do not refer to specific countries. Though there is a dearth of evidence regarding the policy analysis of Uganda’s FHP, particularly the implementation phase, there is limited application of implementation science approaches in the identification of barriers and facilitators [22–24]. Further, despite the importance of health policies in shaping health systems, policy implementation science remains underutilized in health policy research [25–27].

The intersection of implementation science and health policy research presents an opportunity to evaluate policy implementation on a range of proximal factors related to the health system [28]. Despite repeated calls to consider systems thinking in health policy and system research, its application has been slow in low- and middle-income countries. Health system thinking analysis considers system interconnectedness and how each element of the system is likely to affect the results of another [29]. A systems thinking lens is relevant for examining health system policy implementation [28, 29].

In addition, in the context of the deadline when the current SDGs end in 2030 [8], it is not only important, but also urgent to conduct a comprehensive evaluation of the implementation progress of the FHP in Uganda to generate relevant information to optimize allocating the limited resources to achieve maximum impact. However, most siloed evaluations have focused narrowly on aspects of the implementation including health workers’ absenteeism [30–32], drug stockouts, lack of medical supplies and the supply chain [33–36], inadequate financing and the increasing OOP expenses [37–40] and health governance [41–43]. Furthermore, despite the optimization to support implementation through several incremental policy shifts, the policy has not achieved its objectives of enhanced financial risk protection in the population.

Additionally, different key stakeholders at national, subnational and local levels have contributed to the FHP implementation over time. To identify and incorporate context-specific factors, it is recommended to include key stakeholders’ views and opinions [44, 45]. Previous FHP analyses explored the perspectives and opinions of stakeholders, but limited the perspectives included [46–50]

Therefore, this study aimed to explore the perceived barriers and facilitators to the implementation of the free healthcare policy, drawing on the experiences of policymakers, policy advocates, policy supporters/influencers, policy implementers and policy beneficiaries who have been involved in the implementation over time.

## Methods

### Design

In this paper, we employed an exploratory qualitative study design [51] to enable in-depth experience sharing and description of the factors influencing the implementation of the FHP over time. Data were collected using semi-structured interviews with key informants (KIs) and focus groups with policy beneficiaries. Perspectives on implementation over time were collected by incorporating questions relating to understanding the policy implementation journey since its inception.

### Study setting

The study was conducted in purposively selected public health facilities which were from eight local governments, with consideration of regional representations. The four regions were each represented by two districts, including Buikwe and Nakaseke from the central region, Pader and Amudat from the North, Kasese and Kamwenge in the west, and Iganga and Mayuge in the east. As the national-level organizations were mainly in Kampala, Kampala Capital City Authority was selected as the ninth district.

The main sampling criteria were the nationally graded “best and worst” (i.e., an annual grading by the Ministry of Health (MoH) applied to hospitals and health centres IVs [52]. We used a 100-point scale and categorized the health performance into four groups: very good (1–25), good (26–50), fair (51–75) and poor (76–100). If a facility met the criteria but was not public, it was excluded, and the next closest public facility was considered. In total, eight public health facilities were selected, including two hospitals and six health centre IVs, with two facilities per region.

### Participants and recruitment

**Fig. 1:**
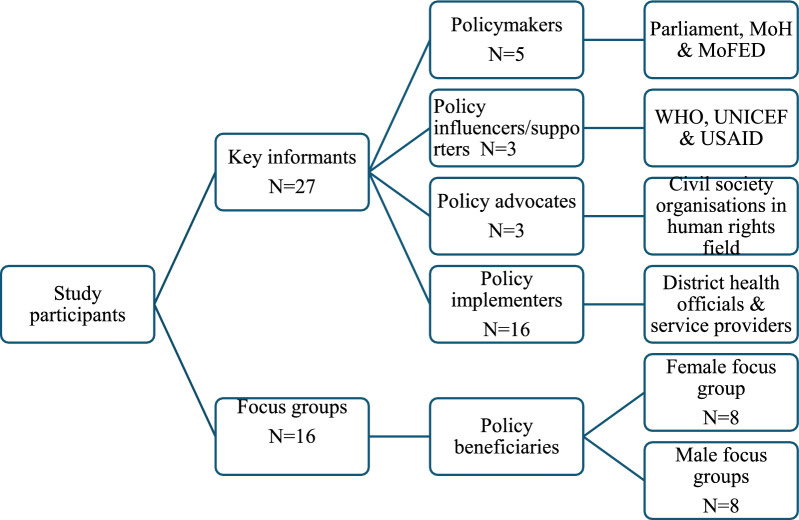
Summary of the study sampling frame

The research focused on 27 purposively selected key informants. At the level of organizations, selection was done with the help of one senior author (F.S.) who has experience in the Ugandan health policy landscape. The selection considered relevant offices and organizational roles in the implementation of the free healthcare policy in Uganda. Key informants were selected if they (i) were experienced in policy implementation for at least 1 year, (ii) possessed knowledge of the FHP implementation and (iii) gave consent to be interviewed. Policy beneficiaries were recruited from around the health facility catchment area, and the inclusion criteria were: (i) aged 18 and above, (ii) had used (were ill or accompanied by a patient) the target healthcare facility in the past 6 months and (iii) consented to be interviewed and audio recorded. Figure 1 summarizes the sampling frame.

### Data collection procedure

Data were collected between May and October 2023. We developed and used pilot-tested focus groups and interview guides. The focus group guides were developed in English and translated into the local dialect spoken in each of the selected districts. Although the interview guide included common questions about the policy of interest, some questions were specific to the participants and the role they played. The lead author conducted the focus groups with translation. Four research assistants experienced in qualitative research were trained in English. The research assistants’ roles were to translate the focus group guide into local dialects, translate during the focus groups and transcribe the interviews. The focus groups were conducted in private places identified by the health facility in charge and approved by participants. Key informant interviews were conducted by the lead author in English. Interviews were conducted in a place of preference for the participant. Five interviews were conducted online (Zoom), and 22 were conducted in person. Interviews and focus groups were audio recorded, and audit trails were noted. Only two interviewed participants refused to be audio recorded, but detailed notes were taken instead. Interviews took approximately 45 min, and focus groups about an hour.

### Data management and analysis

Pseudonymization of key informants was done by the lead author. Interviews were transcribed verbatim in English according to the participant category by the research assistants, and the lead author, using the audio recordings, checked the transcriptions. The focus groups were professionally translated into English by the research assistants and translators. The translation was conducted per focus group, and files were stored with the same numbering as KIs.

Deductive thematic analysis was used to analyse transcribed data. We applied the Consolidated Framework for Implementation Research (CFIR), an implementation science framework described as a powerful tool that enables retrospective evaluations and provides categories for deductive analysis [22, 53]. The framework was used to catalogue, structure and analyse the barriers and facilitators to free healthcare implementation. Damschroder and colleagues (2022) updated the CFIR domains, construct names and definitions (https://cfirguide.org/constructs/). The framework was chosen because its five domains influence implementation, and previous studies have extensively applied it as a pragmatic guide in evaluating intervention barriers and facilitators. Moreover, the framework was found appropriate since its constructs capture complex and multilevel implementation [53], which resonates with the FHP implementation. A summary of the CFIR domains used in this study is outlined in Table 1.

**Table 1:** CFIR domain operational definitions

CFIR domain	Operation definition
Innovation domain	Perception (positive or negative) of the policy design, relative advantage and knowledge of how it works
Outer setting	External support (e.g. funding, donations, expertise) to implement the policy and national-level support and influence on the policy implementation (positive or negative influence)
Inner setting	The “features of structural, political, and cultural contexts through” which the policy is being implemented and their positive or negative influence
Individual domain	Champions for the implementation of the free healthcare policy
Implementation process	Procedures that attract and encourage or deter the participation of different stakeholders in implementing the free healthcare policy; procedures chosen and operationalized, and implementation strategies to address barriers, leverage facilitators and fit the context

*Source: *[22]

Data were coded both manually (16 focus group transcriptions) and using NVivo V.14 software. Since most of the users’ data were collected in the local dialect; research assistants translated the data into transcriptions, and the validity of the transcriptions was ensured by separately hired translators. The transcriptions were manually coded by P.K.N. This helped P.K.N. to understand the insights in the data since the coding process enabled reading and rereading the transcripts to become familiar with the data. Focus group coding was done using highlighters and memo’ing in the page margins. To identify gender-specific perspectives and experiences, the coding of focus group responses was separated for men and women. Nvivo was used to aid data organizing and analysis, applying deductive (CFIR) and inductive coding. P.K.N. deductively coded all the transcripts, and C.C. independently coded 20%. P.K.N. and C.C. met to compare and discuss the codes and resolve discrepancies by reaching a consensus, improving inter-code reliability [54]. Initial codes were generated guided by the CFIR domains/constructs, and each transcript was coded after the coders familiarized themselves with the data by reading the text several times. Codes were grouped into subcategories and themes, whilst similar codes were merged. They were later organized according to the UHC indicators. Data triangulation was conducted by comparing codes and themes firstly across the gender focus groups and secondly with and across the different key informants. The secondary analysis compared themes across the CFIR domains and participants’ experiences, perspectives and perceptions at national, subnational and health facility levels.

Table (Table – Supplementary) provides study participants’ synthesized experiences of the factors that influenced the implementation of the FHP. Whilst we used the CFIR framework to organize the factors in the analysis, the study questions and the sampling framework were not guided by the framework. As such, some sections of the CFIR constructs had no identified factors. To organize and categorize data systematically, we developed a coding scheme that mapped data to specific CFIR constructs. We first coded focus groups (according to gender), followed by key informants’ interviews (according to category), and later compared data within and across these two broad groups to understand common facilitators and barriers. The codes were classified at national, subnational and health facility levels to determine where the factor was being experienced most. The analysis and interpretation of the coded data looked at how constructs interacted and influenced (positively or negatively) the implementation. The coded facilitators and barriers were mapped and aligned to the UHC indicators of service coverage, financial protection, and equity to draw action insights regarding UHC. Applying interpretive analysis, we articulated that the overarching contextual factor was inadequate resources.

## Results

### Demographics

Data were collected from eight public health facilities. Two were at the hospital level, whilst six were health centre IVs. The 16 focus groups had 97 participants, with 51% being female. A total of 27 key informants participated in the study, and the majority (70%) were male (Fig. 1).

### Barriers and facilitators influencing the implementation of the free healthcare policy

Five factors were synthesized and categorized as barriers and facilitators, with some overlap between the five factors: (i) financial resources, (ii) medicines and supply system, (iii) health human resources, (iv) infrastructure and functionality and (v) equity and the FHP Implementation. In the sections below, we describe the policy implementation changes that relate to each factor and discuss the findings on how the factor presented a barrier to implementation, supported by participants verbatim, followed by facilitators in the same manner. In this paper, the perspectives of participants are presented in a brief of the historical changes within each factor, the description of the barriers, and where possible, in participants’ direct quotations with a focus on the time horizon. Overall, data show a strong alignment between the five factors reflected in the perspectives, perceptions and experiences of the participants.

#### Financial resources

Key informants noted that the President of Uganda abolished user fees in public facilities (except hospitals’ private wings) in 2001, shifting the health sector financing responsibility to the central government through general tax revenue. However, since the inception of free healthcare service provision, health services have been financed through a combination of government revenue, out-of-pocket (OOP) payment and donor funding. All key informants noted government under-funding of the health sector over time, making the sector rely on private sources. Consequently, users have continued to pay for some services that were meant to be free. The National Health Insurance (NHI) agenda, which is expected to bring extra domestic income to the health sector financing, has been on the political agenda for more than two decades. Participants noted such financial resources factor:

I recently plotted a graph, and I was really surprised when I saw our out-of-pocket (OOP) payments. I noticed that from around 2000, when the reform had just happened, we had the highest amount of OOP expenditure on health. This has later reduced, but it has spiked in the last decade. So, that shows you a clear view that the policy had a reverse of what it is to achieve (Policy Supporters #1).

For example, health insurance has been on the agenda since 1987. Now we are almost 40 years old, we haven’t done much. And there is no hope (DHO #5).

The financial resource factor was raised amongst all key informants. Insufficient fund allocation through the national budget to the health sector and the delays in releasing finances by the Ministry of Finance, coupled with inefficient implementation, were cited as major reasons behind the health sector’s financial constraints. Inefficient implementation was observed in terms of less investment in primary healthcare (PHC), including prevention activities. Health professional participants explained that the most common causes of morbidity and death were preventable diseases such as malaria, respiratory infections, neonatal conditions and vaccine-preventable diseases. Although additional funding was made available by external donors, who provided more funding than the government, donor funds came with constraints that hindered implementation. Inflexible grant conditions, report formats and periods prescribed by donors were hard and burdensome for officials, who, as a result, returned the funds to donors. The outcome is the increasing OOP payments that are sometimes catastrophic and have persisted for more than two decades of policy implementation [11, 12, 38, 39].

Additionally, the President’s political will towards the policy did not translate into sufficient allocation of funds to the health sector. The interviews revealed that the president and ministers are part of the Cabinet that allocates the national budget; the Parliament majority are members of the ruling party, and Parliament appropriates the national budget, but their political will is unrealized given their lack of allocation to support the commitment.

Examples of what participants expressed as financial resource barriers:

The problem we have with donor money, like global funds, the World Bank and GAVI, is a low execution rate, sometimes as low as 40%. This challenge is the reporting systems and the difference in reporting periods. That is, they will not release the money until you provide accountability for the last quarter (Policy Supporters #1).

We engage the Parliamentary Health Committee and officials from the Ministry of Finance to push and make sure that yes at least, allocate more money to the health sector. It is not an easy thing. We have been there for the last 4–5 years, but I want to tell you the increments are very small (Policy Advocates #1).

…. inadequate finance, much as the goodwill is there by our leaders, the financing is inadequate, and it’s been the situation since inception (Policymaker #2).

There is less financial investment in preventable and public health activities, yet it is evident that spending on such interventions significantly reduces national healthcare spending in treating such diseases, saving money to finance other aspects of the system (Policymaker #3).

Public participation in budgeting, as witnessed through engaging stakeholders in the planning and budgeting process at all levels, was identified as a facilitator in policy implementation. The budgeting process was noted to be bottom-up (starting at health facilities), and key stakeholders at the health facilities, districts and ministry are engaged. Additionally, external financial support is received from different external donors, some of which are focused on PHC. The support was mainly for human immunodeficiency virus/acquired immunodeficiency syndrome (HIV/AIDS), tuberculosis (TB), maternal and child health. National-level participants articulated that:

Nearly all the development partners have a global strategic plan for PHC. GAVI and the Global Fund also have strategies around PHC and pandemic preparedness (Policy supporters #2).

The health budgeting process is participatory and drawn from the health facilities upwards, so actual priorities are budgeted for (Policymaker #3).

#### Medicines and the supply system

Participants noted a positive policy shift across most segments of the supply chain, leading to a stable medication supply. National Medical Stores (NMS), a government-owned institution, distributes medical products to all public health facilities, and it has offices at the national level in central Uganda. At the policy introduction, a kit system (pre-packed pharmaceutical ration kits containing an assortment of medicines and medical supplies designed to supply a given number of patients) was operated for essential medicines supplied quarterly and this was later replaced with the pull system (health facilities request medicines and medical supplies on the basis of demand) except for health facilities at level II and III. Currently, NMS follows a schedule to supply medicines every 2 months to health centres IV and hospitals through last-mile delivery, where medicines are delivered to the district health office (DHO) first and later distributed to healthcare facilities. The health facilities place their orders for distribution, and NMS delivers them. One policy implementer said that:

What I have seen is that the shift of policy for most of the supply chain has changed positively and progressively. The two-month cycle and the introduction of the last-mile distribution have improved the supply chain. NMS took the responsibility of first storing the medicines at the district, then from the districts, delivery is made to specific health facilities, and the health facility acknowledges receipt (DHO #3).

However, chronic medicine stockouts at public facilities have been perceived by all participants from the policy’s inception to date as a barrier impacting implementation. Inadequate budget allocation, delays and changes in delivery by national medical stores (NMS) were the three major reasons for the stockout. Providers indicated a mismatch in the continued increase in the population served over the years and budgets allocated for essential medicines. Delays of 4–6 months and the variation in what was delivered versus what was ordered were cited across all districts. In the recent past, NMS had started using a delivery schedule, but it was not followed judiciously, thus the delays. After the kit system adjustments, each facility is allocated a budget for medicines and medical products, and healthcare providers send in their requests according to the money they have. In instances where the budget is depleted before the end of the year, NMS cannot “loan” out drugs to such facilities. Two policy implementers and policy beneficiaries explained the persistent challenges in the medicine and supply system:

….the supply by the NMS is so irregular, and that is how it’s been from inception. With the changes, we are supposed to be getting medicine every 2 months. We have six cycles in a year, but up to now, we are still demanding one cycle of the last financial year (May–June 2023), which has not yet been delivered. But even the one for this financial year has not yet been delivered (DHO #7).

When you look at the absolute figures, the medication and health supplies budget has been increasing. But this increase caters to inflation, and it’s not aligned with the increase in the population we serve. But if you look at the policy trajectory, the budget for medication, and the increase in population, this budget has been static.

(Health provider #5).

You will never find medicines in this health facility; you must always go and buy them. It’s free healthcare, but you do not get free services (Focus group female #6).

However, in the last decade, ways to overcome challenges relating to the medicines supply chain systems have been reformed. First is the introduction of the last-mile delivery system. The system allows the delivery of medicines to health facilities directly to where the health facility in charge and one member of the community acknowledge receipt. Second is the redistribution of medicines and products. District healthcare systems use WhatsApp groups as their communication networks to express surpluses and deficits and enable medicine to be redistributed. One policy implementer explained the strategy of redistributing medical products within health facilities:

With the introduction of the “Redistribution of Essential Medicines and Health Supplies”, we are tasked to redistribute drugs from where we have a surplus to where they are lacking. We only get gaps after the redistribution has been exhausted…Like now for antimalarial, we asked on the platform who had, and everyone said they did not have it (Health provider #3).

#### Health human resources

All key informants reported concerns about staffing norms that had not changed for more than two decades, until recently (2022) when a new structure was developed by the MoH and shared with districts. Staffing norms refer to the normative statement indicating the required number and skill mix for a given health facility [55]. By the time of the study in late 2023, the new guidelines had not been operationalized in all districts. Inadequate funding was stated as a constraint. Policy implementers and makers confirmed that the human resource structure has not changed since the policy’s inception:

…the staffing norms that were in place 27 years ago (since I joined the service) have remained the same. Except for medical officers, where they introduced special grade medical officers, but the nurses, midwives, etc., this has never changed (DHO #2).

Over time, the … human resource structure …is the challenge; the disclaimer was that the structure is subject to the availability of funding, which is why even the recent changes have not been implemented (Policymaker #1).

The staffing norms have continued to be the same for more than two decades and have contributed to the shortage of health workers, and this factor was raised by all participants. All the health facilities were understaffed, having only 60% of the necessary staffing levels (number of current employees) even for healthcare cadres such as nurses and midwives, whose production has greatly increased over the decades. Besides the staffing norms that were not revised for more than two decades, the shortage was also due to the ban on the recruitment of staff and the increasing population, resulting in the demoralization of the few health workers. In some districts, the situation was worsened by governance challenges where the health service commission (in charge of recruitment to replace those who retired, left or died) was not constituted for 2 years, and as such, recruitment was not conducted. In addition, in some districts, the appointment to the commissions was more based on political rewards and not competence, causing a rift between the political and technical wings of the districts. The shortage resulted in an increased workload for health workers and long waiting hours for users. The low staffing norms were also evident at the district health office, and participants confirmed this:

At this facility, staffing is still poor, it is only about 52% (Health provider #3). Now, as we talk at this health centre IV, we have 10 staff, including the support staff, for 12 years. If you remove them, we are six healthcare providers (Health provider #8).

As far as I know, the free healthcare policy has been characterized by a continuous massive shortage of human resources for decades. For example, Kawempe National Hospital has a staffing level of 35% (Policymaker #7).

Though the shortage of health workers emerged as a barrier, policymakers noted a continuous and steady increase in the production of all health cadres. Specifically, the overproduction of nurses and midwives was cited. Users observed that health workers in public facilities were better trained and more experienced compared to their counterparts in private facilities. Consequently, despite the inherent challenges within public facilities, users consistently opted for those services. Participants noted the following human resource factors:

We continue to come here despite delaying receiving services because there are few workers and a lack of medicines. However, unlike private facilities, government facilities have trained staff. So, you come, and at least they write for you the medication (Focus group, male #6).

Over the years, we have seen steady progress in the production of all cadres of the health workforce, and this keeps increasing even within previously scarce cadres like pharmacists (Policymaker #1).

#### Infrastructure and functionality

Policymakers and implementers at the subnational level noted that over the study period, there has been increased investment in infrastructure, particularly in building and renovating health facilities, leading to reduced geographical access barriers for people seeking health services. In addition, in 2016, the government announced the plan to phase out health centres II by upgrading them to III, and it has since embarked on funding this course. Policymakers and implementers noted these positive incremental changes:

We have seen a steady increase in the number of public health facilities since the introduction of the free healthcare policy. I might not have the exact figure, but in this district, the number has almost doubled since 2001 (DHO #2).

I have been in Parliament for two terms now, and since the government started to upgrade health centres IIs, every year we appropriate funds to that vote (Policymaker #5).

The infrastructure and its functionality factor were pronounced amongst healthcare providers. Not all the health centres IV were fully functional because they were not equipped. Notably, the surgical equipment in the theatres was missing; thus, operations and blood transfusions were not conducted. Four out of the eight health facilities had ambulances, but only one was functional, and in some facilities, simple equipment such as beds were insufficient for the volume of patients, whilst many were in bad condition. In addition, in situations where the equipment broke down, repairs were not conducted on time, further constraining service delivery. The design of the health facilities was supposed to provide accommodation for the staff within the facility premises; however, all of them have inadequate accommodation for house staff. The alternative was rentable houses, but in some settings, they were distant from health facilities. All these challenges arose because of insufficient funding. Policy implementers mentioned that:

The challenge is theatre equipment, and we have no equipment for blood transfusion since the health facility was started 8 years ago. So, we do not conduct things like a C-section (Health provider #7).

We are using available resources, but we need the expansion of the structure, and also, we need instruments and equipment. Since 2000, nothing new has been bought amongst the major equipment. (Health provider #2).

I can give an example here, infrastructure such as houses for the staff, we have no houses for the staff. We need more wards. An example I can give you is that we have that maternity ward, and it has 10 beds, and yet we are supposed to have 16 beds (Health provider # 5).

The existence of governance and management structures at health facilities and district levels was noted as a facilitator. It was uncovered that these governance and management structures existed before the FHP but were formalized and strengthened after the policy was introduced. Each health facility level has a unit management committee as a key governance structure with representatives from staff and the community. The roles of the committees, amongst others, included the promotion of transparency in materials and resources, exchange of stakeholder feedback, advocating for improved quality service delivery, monitoring the general administration of the facility and approving annual budgets. At the district level, the district health management team (DHMT) includes health facility managers within that district, representatives from private healthcare providers and local NGOs, members from other district health departments, administrative district managers and political leaders. Their major role is ensuring coherence with national policies as they oversee the implementation of health services. Another an important facilitator was continuous investment in building health facilities. The central government, through PHC development grants, has continued to invest in building more government facilities and recently upgrading and renovating existing ones to improve service delivery. Additionally, the central government sends other PHC grants to the districts to facilitate service delivery. Policymakers noted infrastructural facilitators:

… putting that aside, the fact is we prioritize allocating funds for upgrading health centres; for example, from HC III to IV at least in each constituency (Policymaker #4).

The number of people within a radius of 5 km has increased from what it was in 2001. We are at 85% now, from less than 50%. I consider this an improvement (Policymaker #2).

#### Equity and the free healthcare policy implementation

Although no distinct equity domains were uncovered from the policy implementation, we found some facilitators of universalism. They were established on two aspects: who was benefitting and how the financial allocations were considered. The policy design and implementation targeted everyone within the country, underpinning UHC’s principle of “leave no one behind”. The financial allocation to districts was done using a resource allocation formula that was transparent and intrinsically promotes equity by ensuring districts receive a fair distribution of health resources. The resource allocation formula variables were based on variables such as poverty, population and topography, amongst other variables.

### Interpretive summary of findings and the framework

Findings showed that perceptions amongst participants at different levels were in unison, echoing that participants had experienced similar results of the policy implementation, irrespective of where they were situated. This validates the accuracy of the raised barriers and facilitators to implementation at different levels. Equity factors in the policy implementation were not feasible, but rather some features of universalism were identified.

The majority of the service coverage facilitators, such as the policy objectives being clear (all services in public facilities are free), political leaders sensitizing people about the free healthcare services, and services being provided by trained personnel, stem from the innovation domain. Barriers include the ban on recruiting human resources, the health human resource structure that has remained unreviewed for more than two decades, medicines and supplies coordination at the national level and drug stockouts, amongst others flow from the inner setting and implementation process domains at the districts and health facility levels. This points to the need to better understand contextual factors and design implementation strategies to fit the context, reinforcing that successful implementation of health policies depends on the context.

The majority of financial protection facilitators, including continued external financial and technical support and the Minister for Health being part of the Cabinet that allocates funds, stem from the outer setting domain at the national level, whilst barriers flow from the outer and inner settings, both at the national level. In addition, the inner setting barriers such as delay in the release of funds, inadequate financing affecting support supervision, delays in repairing equipment and some places having no health facilities that can provide secondary care, altogether have a visible effect on health facility service provision. The financial protection barriers enforce the gap between health policy intent and implementation, normally stated as “policies are not always implemented as envisioned” [1, 2]. The role played by the policy champion (President) from 2001 to date and the implementation lead (Minister for Health) in ensuring sufficient funding for policy implementation is not visible. Their role did not translate into securing sufficient funding to enable policy implementation, as the health sector has been chronically underfunded.

The categorization of barriers and facilitators using the UHC indicators illustrated that although financial barriers arise from the national level, they directly impact and affect service provision at subnational and health facility levels. Barriers relating to service coverage mostly stem from insufficient funding, whilst barriers relating to financial protection relate mainly to increasing overall government allocation and efficient investment in public health activities. This reinforces the connectedness between service coverage and financial protection of the UHC indicators.

## Discussion

Drawing from policymakers, policy advocates, influencers, implementers and beneficiaries, this study provides insights into the key facilitators and barriers that impacted the implementation of the FHP in Uganda from 2001 to 2023. The policy implementation gaps are explained by limited resources, political will that does not translate into sufficient allocation of resources for the FHP and complacency with incremental policy shifts that are not driving meaningful improvements in the health system. The study reinforces the impact of contextual factors and forces working against and for the implementation of free healthcare policies in limited resource settings. The identified barriers and facilitators were categorized using the CFIR domains [22]. Applying CFIR was an effective way to analyse the data, aligning well with the implementation science understanding of barriers and facilitators to policy implementation. Although adaptive, in the context of this study, some constructs were repetitive, for example, local conditions and funding as a resource, whilst others were not very relevant, such as market pressure and relative priority, reiterating the need to purposively select constructs that apply to a project.

We identified five major factors deemed significant barriers and facilitators, four of which are resources. The participants described that resources were fundamental for the successful implementation of the policy. Insufficient financing significantly impacted the human resources, health supply chain system and infrastructural inputs, reflecting the interwoven and interdependent nature of components of the health system. Insufficient financing is a result of inadequate domestic allocation to the health sector, coupled with delayed releases of funds and inefficient implementation. Since the policy was enacted in 2001, the money allocated in absolute terms has been increasing, but not in parallel with the increased population and inflation. Despite the population of Uganda having almost doubled from approximately 25 million in 2001 to 46 million people in 2023 [56, 57], the health sector budget allocation has not been commensurate. For example, current health expenditure per capita steadily increased between 2003 and 2011 before stagnating up to 2014, and has since then continued to decrease [58, 59]. The benchmark as the minimum for a generic essential package in low-income countries such as Uganda is estimated at USD34 per capita, but Uganda’s spending has stagnated between USD11–15 per capita for years [6, 60]. The funding constraints are expressed as systemic and systematic, and they affect supportive services provided by the MoH and service delivery within the districts [60, 61]. Despite the abolition of user fees in 2001, Uganda’s healthcare system continues to depend significantly on OOP payments as a source of funding [18, 62–64]. Excessive reliance on OOP payments hinders the attainment of UHC by constraining access to critical healthcare services, creating financial hardships and exacerbating poverty, especially amongst vulnerable populations [65–67].

The results indicate that insufficient funding affects the inadequacy of inputs, yet most donors are reluctant to contribute to items such as human resources salaries [68]; donors pivot their investments on essential medicines and health supplies (EMHS) that focus on specific vertical disease programs such as immunization, malaria, HIV/AIDS, and tuberculosis (TB) [35, 69]. A shortage of health workers is a critical barrier to service delivery. Staffing norms were not revised for more than two decades, support supervision by the district officials was not effectively done by the DHOs and the ban on the recruitment of health workers manifested in the wage-bill ceiling [70], resulting in understaffing and over-burdening of the available staff impacting the quality of health service delivery [71, 72]. Health staffing was unmatched by population growth due to staffing norms that were not revised for more than two decades until recently, making the country fall short of the WHO recommendations to realize UHC [49, 73]. Another input most affected by inadequate funding was the health supply chain system. The key cause of the chronic EMHS stockouts was low budget allocation, considering the country’s health needs [35, 69, 74]. The policy beneficiaries were, in most cases, asked to buy medicines due to stockouts, implying financial protection was not fully achieved. Previous studies have shown that out-of-pocket payments for outpatient medicines account for the highest share of health spending amongst individuals [67, 75], making it a key driver for financial hardship and unmet needs for families [67]. The results indicate that there was relative under-investment in basic infrastructure inputs. Financing health infrastructure – particularly staff accommodations and health facility equipment – is a long-term investment critical to improving public healthcare access, quality and efficiency [76–79].

The findings illustrated that the political will does not translate into sufficient allocation of funds. Participants noted that the president, prime minister, minister for health and many other senior officials both at the national and local levels make public declarations with no connection to concrete action. Despite the FHP having strong political support, the allocation to the health sector is still insufficient, below the Abuja declaration on health where African Union governments committed to allocating at least 15% of their national budget to improve healthcare [60]. Additionally, the National Health Insurance (NHI) scheme has been on the agenda for decades [80]. Although the insurance scheme is an avenue to mobilize more funds for the health system, the establishment efforts have been static. Evidence in a similar setting shows that political will is critical in bringing about big health reforms [81–83]. The president has never officially supported nor blocked the proposal to start the NHI scheme [81]. However, in March 2021, the then Minister for Health attempted to withdraw the NHI bill from the floor of Parliament. Although Parliament objected to the request and passed it into an Act of Parliament, it was recalled and is pending reintroduction to the 11th Parliament for fresh discussion [82]. Evidence shows that dedicated public spending is the tangible demonstration of prioritized political will [83, 84]; in the same way, economists such as Joseph Schumpeter pointed out that “the budget is the skeleton of the state stripped of all misleading ideologies” [85]. Although the political will is necessary to ensure appropriate levels of funding, the study found it insufficient.

Additionally, inadequate domestic fund allocation implies that the government relies heavily on external donations. The results indicate that external donations come with conditions that are burdensome to the relevant officials, resulting in less than 50% utilization of their funds, despite the funding gaps. Further, instead of weaning away from over-dependency on donors, the Ugandan government is increasingly receiving more donor funds by the day [59, 61]. Given that Uganda is a low-income country, it is perennially unable to fully finance its public health system [86]; as such, additional resources from donors boost the health system’s financing. Ironically, since 2007, external funding seems to be displacing domestic funding, as evidenced by decreases in domestic government financing as external funding increases [87]. Extensive evidence shows that external assistance is not without problems; notably, undermining domestic funding and decision-making and reducing local allocation for health and accountability, amongst others [88, 89].

Over time, several incremental changes were noted in implementation, including in the supply chain, a focus on the production of PHC health workers, governance and management of the district and health facilities and health infrastructure. Although the marginal adjustments were to prevent the policy from drifting into failure, results uncover a persistent pull of the status quo [90]. For instance, the implementation of last-mile delivery, harmonized distribution and in-district redistribution [35, 69, 91] offers the opportunity for sustainable medicine use. Other government efforts to improve the medicines and supply system include: the Buy Uganda, Build Uganda policy, which has increased the production of pharmaceuticals, locally mitigating the risks associated with importing medicines; the national formulary and national medicine register to rationalize the medicine selection built on essential medicines and health supplies list (EMHSL), which enables the optimal management of set resources and the increased availability of life-saving medicines; and pooled procurement of medicines and medical products in the public sector by NMS [91–97]. Nonetheless, participants at the subnational and health facility levels and policy beneficiaries decried chronic drug stockouts since the policy’s inception to date. Evidence shows that the status quo has a great role in structuring responses to proposals for policy change, resulting in ignoring comprehensive alternatives, considering them unpredictable in their consequences and impractical in political requirements [90, 98]. Aligning with the supply chain incremental changes, reforms were focused on the supply side (NMS), ignoring drug stockout bottlenecks stemming from the health facilities, including expiration of commodities, poor inventory management, poor storage and management and ineffective supervision and oversight, amongst others [35, 69]. Future research could analyse some of the free healthcare policy incremental choices that have diverged from the status quo and led to a positive drift.

However, for successful policy implementation, research has long pointed to the importance of involving policy implementers from the onset of the policy process [99]. This FHP was a quick reform, not allowing time for consulting key stakeholders, including healthcare providers. In addition, the role and importance of context in the success of health policy implementation have been found in previous studies [100–102]. The analysis explored the main contextual factors that significantly affect the FHP implementation, and as such, builds on that evidence. Additionally, financial protection facilitators arise from external support from donors and national-level support and influence. The main revenue sources for financing healthcare have for years been topped by household out-of-pocket costs, followed by external sources, and lastly, domestic funding [61, 103]. Free service provision requires money, and in the initial stages of the policy implementation, the government increased health workers’ salaries, health facilities and essential medicine budgets [7, 103, 104]. Similar to what was found in previous studies [7, 103], the increase was not sustained in the long term, indicating that political leaders are not “funding the promise”.

Nonetheless, although no document detailing the FHP exists as there is no formal approval of the policy by the Parliament and President [7, 103], we found that all participants had sufficient information about the policy. It is a financing policy with a universal free care benefit package in public health facilities [6, 7]. It is not surprising that service coverage facilitators arise from the perceptions of the policy design, its relative advantage and knowledge of how it works (innovation domain). Indeed, evidence shows that Uganda’s service coverage index doubled from 22% in 2000 to 50% in 2020 [6, 59, 60], indicating progression on UHC indicator 3.8.1. However, several other factors might be attributed to this success. For instance, the donor aid through off-budget has consistently and for decades been invested in specific aspects of the UHC service index relating to child vaccination, HIV/AIDS, TB and maternal care, thereby sustaining high-level financing for such services [35, 60, 61, 69, 105]. This is coupled with the steady increase in the production of health workers, particularly nurses and midwives who are at the front line in providing those services [73, 106].

### Policy and practice implications

Evidence shows that countries have succeeded in moving away from over-dependence on development assistance to sustainable financing of their essential services using domestic resources, and it started with health system investment [107]. Alternative funding sources will contribute to weaning away from donor dependence; development partners should focus on supporting and guiding Uganda to develop “home-grown” health financing initiatives. Ugandan political leaders’ commitment to the FHP needs to be commensurate with allocating the health sector the minimum required resources (e.g., 15% of the national budget as per the Abuja declaration). Policy advocates need to step up advocacy efforts to increase domestic allocations for health. This could be done by providing alternative innovative ways of increasing health financing such as sin taxes and tax credits [108, 109], which have been found effective when the generated revenue is earmarked for specified health-related spending; and by supporting efforts to start the NHI scheme as a means of mobilizing funds from policy beneficiaries. The ruling government has twice (in 2011–2016 and 2016–2021) reflected NHI in its manifesto but has never delivered on that promise/policy [80]. Just as the president seized the window of opportunity during the presidential campaign and removed user fees [7, 110] to have a quick policy reform, policy beneficiaries could use a rare window of opportunity for the president’s campaigns to propel the candidates to commit to increasing health sector allocation and/or establishing a NHI scheme. Evidence from Ghana, Thailand, Sierra Leone and Rwanda supports this approach [79–81, 111–113]. Although generating and allocating more funds to finance the health sector is crucial, efficient use of the available resources is required. Inefficient policy implementation could be addressed by MoH officials, creating innovative ways of encouraging health promotion actions amongst policy beneficiaries and healthcare providers [114]. Since such decisions are taken in social and political contexts, MoH officials need to articulate the opportunity costs clearly before introducing any resource-intensive intervention. Several relevant health technology assessment tools exist and are applicable in many contexts [115].

### Reflexivity and positionality

The lead and one senior author have lived experiences with the Ugandan healthcare system. The other authors have engaged in several health research studies in Uganda, qualifying us as insider-researchers [116, 117]. Although none of the authors has ever engaged in a scholarly assessment of barriers and facilitators of any healthcare policy in Uganda, having prior interaction and experiences with the healthcare system might have resulted in preconceived ideas regarding the challenges the Ugandan health system faces, thus having biases during the analysis and interpretation of results. A neutral author (C.C), the qualitative researcher who has never interacted with the Ugandan healthcare system and not engaged with health research conducted about Uganda, was involved in data coding, analysis and interpretation of the results.

### Limitations

Qualitative research poses some limitations, including recall bias and purposeful selection of participants. An unconscious bias may have affected the study outcomes, owing to the participants’ and researchers’ experience regarding FHP implementation. For instance, many participants could not recall in detail or accurately the major reforms/policy incremental shifts and turning points in the implementation process, implying we might have missed out on some important historical details. However, recall biases were minimized by providing cues for major changes and dates. Participants in focus groups were conveniently sampled, which may have led to the exclusion of potential participants. However, the inclusion criteria were detailed, emphasized relevant experience and none of the participants included are considered a vulnerable population. Although we assured confidentiality and anonymity to all participants, health professionals may have constrained the expression of their views, not wishing to give negative responses about their work or their employer. However, data source, method and research triangulation were conducted to enhance the credibility and validity of the findings. In addition, the recruited sample participants were diverse in terms of profession and roles, enabling inclusive insights and data triangulation, and we found strong alignment in the views of the participants.

## Conclusions

This study examined the experiences, perceptions and perspectives of policymakers, policy advocates, policy influencers, policy implementers and policy beneficiaries to examine the barriers and facilitators to the implementation of the FHP. The study contributes to the limited literature regarding the scale-up of free healthcare policies in low-resource settings by providing insights into barriers to the successful implementation of FHP. Using the CFIR and UHC indicators, the overarching contextual factor was analysed as inadequate resources; specifically, insufficient funding, which impacts the human resources, health supply chain system and infrastructural inputs. Understanding the connection of these barriers is worth considering to be effective in ensuring Uganda’s progress towards UHC. The insights from key stakeholders can help inform similar settings on experiences in scaling up free healthcare policies in support of UHC goals. Going forward, policymakers need to give special attention to addressing obstacles to the implementation of the policy, including considering strategies to scale up policies such as domestic financial investment. Future qualitative work on FHP implementation should transparently assess each of the incremental changes throughout the years.

## Supplementary Information

Additional file 1.

## Data Availability

Our data consist of the participants’ responses to interview questions. Due to the high-level status of our participants as managers, bureaucrats and policymakers, data can be accessed from pk.namyalo@mail.utoronto.ca through a formal request for research purposes.

## Abbreviations

CFIR: Consolidated Framework for Implementation Research
EMHS: Essential medicine and health supplies
DHMT: District health management team
DHO: District health officer
FHP: Free healthcare policy
KIs: Key informants
MoH: Ministry of Health
NGO: Nongovernmental organization
NHI: National health insurance
NMS: National medical stores
SDG: Sustainable development goals
OOP: Out-of-pocket
PHC: Primary healthcare
UHC: Universal health coverage
