# Supplementary material for: Exploring the facilitators and barriers to achieving universal health coverage in Uganda: a qualitative study of the free healthcare policy
Source: Health Res Policy Syst. 2025 May 19;23:60. doi: 10.1186/s12961-025-01334-8 (PMC12090541; doi:10.1186/s12961-025-01334-8)
Supplement: Supplementary file 1 — Additional file 1. [file 12961_2025_1334_MOESM1_ESM.docx]

**Supplementary**

**Table: A visualization of the UHC Indicators and CFIR domains that emerged as facilitators and barriers for free healthcare implementation.**

| **UHC indicator** | **Experience** | **National level** | **Sub-national** | **Health facility** |
| --- | --- | --- | --- | --- |
| **Service Coverage** | | | | |
| 1. Innovation domain (Perception of the policy design, relative advantage, and knowledge of how it works) | Facilitators | 1)The policy objective is clear (all services in public facilities are free)  2)Clear feedback channels (e.g., annual joint review meetings) | 1)Political leaders sensitize people about the free healthcare services | 1) Services are provided by trained personnel  2)Clear feedback channels (e.g., suggestion boxes, community dialogues) |
|  | Barriers | 1)Broader aspects of the benefits package had not changed (Less emphasis on NDCs, in principle, all services are free) | N/A | 1) Reduced trust in vaccines – provided during mass immunization (cultural and religious beliefs)  2) 3) Have no alternative facilities nearer to us  3)People know and appreciate the policy objectives |
| 1. Outer Setting (External support (e.g., funding, donations, expertise) to implement the policy and national-level support and influence on the policy implementation) | Facilitators | 1) MoH plays a critical role in the implementation (Provides guidelines, clinical, allocation, and management of finances, prioritizes hard-to-reach areas, donor mapping) | N/A | N/A |
|  | Barriers | N/A | 1)Inadequate consultation of implementers (e.g., Contracting at the center and implementation at the district, Upgrading of health HC IIs to HC III) | N/A |
| 1. Inner Setting (The “features of structural, political, and cultural contexts through” which the policy is being implemented) | Facilitators | 1)Staffing norms have been revised, but are not operational yet | 1) Redistribution of medicines and supplies  2)The last mile system started by NMS is improving the supply of medicines  3)The supply schedule is every two months | N/A |
|  | Barriers | 1) The ban on recruitment (staffing levels, long waiting hours, …..)  2)Staffing norms have not changed for over 2 decades  2) Districts can only make recommendations – no major reforms in the policy  N/A | 1) Most DHOs have been acting for years (10+)  2) Medicines and supplies are coordinated at the national level (NMS)  3) Less functional referral system – poor communication network | 1) Users go for curative/treatment and never target prevention services  2)The NMS system is stringent and chronic delays in deliver  3) Facilities not providing required healthcare services  4)Drug stock-outs |
| 1. Individual domain (Champions for the implementation of the free healthcare policy) | Facilitators | 1)The presence of functional government structures including technical working groups | 1)Ruling party leaders are the majority at all levels and popularise it  2)Presence of government structure (DHMT) | Presence of governance structure (UHMC) |
|  | Barriers | 1)The champions and the affluent don’t use the services – fly out of the country for treatment | N/A | 1)Implementers were never consulted (Political – limited buy-in on free services) |
| 1. Implementation Process (Attract and encourage the participation of different stakeholders in the implementation of the free healthcare policy; choose and operationalize implementation strategies to address barriers, leverage facilitators, and fit the context | Facilitators | 1) National level – technical working groups | 1)District level – DHMT | 1) Health facility – health unit management committees and community dialogues  2)More nurses and midwives trained |
|  | Barriers | 1)Key performance indicators are curative outcomes  2) Inefficiency implementation (30% of OPD cases are malaria, but no residual spraying due to potential harms, Less investment in road traffic accident prevention – the cost of treating one case is unbelievable | 1)Stakeholders not attending meetings (district and users during community dialogues)  2) Inefficiency implementation (Adolescent pregnancy prevention, NCDs, RTA, etc)  3)District health service commissions play politics (not operational) | 1) The capacity of community representatives in the health unit management  2)Understaffing coupled with the increase in population  3)Lack of ambulances to transport patients  4)Have no required equipment to provide the required services |
| **Financial Protection** | | | | |
| 1. Innovation domain | Facilitators | 1)The policy objective is clear (all it’s a financing policy in public facilities are free) | N/A | 1) All services are free at the point of access |
|  | Barriers | N/A | N/A | 1)Free healthcare services are free of services  2)Cannot afford to pay for healthcare services |
| 1. Outer Setting | Facilitators | 1) External support (over 40% of health expenditure) and technical support – PHC  2) MoH part of Cabinet – allocation of funds, and the Parliament committee has a chance to discuss the budget |  |  |
|  | Barriers | 1)Inflexible grant conditions and report formants  2) NCDs are not a priority  3) Allocations by the Cabinet can only be adjusted up to 1% in Parliament  4) Inadequate accountability by the center | N/A | N/A |
| 1. Inner Settings | Facilitators | 1)PHC development funds to districts and for service delivery. | 1)The planning and budgeting process is bottom-up  2)GoU-released funds are utilized | 1) Investment in building more health facilities  2) GoU-released funds are utilized |
|  | Barriers | 1) Delay in the release of funds from the central treasury  2) Increased population Vs funding  3) Inadequate financing affecting supervision (for DHOs)  4)The inflexibility of the money - comes earner marked and cannot change it | N/A | 1) Increased disease burden Vs funding  2) Disincentives for health workers (staff accommodation, no promotion, ……)  3) Equipment breakdown and repairs delay  4) Some places have no health facilities to provide secondary and tertiary care |
| 1. Individual domain | Facilitators | It was a presidential decree in 2001, still in power, and strongly supports the policy | N/A | N/A |
|  | Barriers | 1)Governance challenges (Change in leadership every 5 years)  2)Slow in introducing the NHIS | N/A | N/A |
| 1. Implementation Process | Facilitators | 1)Increased investment in building and renovating health facilities | N/A | N/A |
|  | Barriers | 1)Inefficiency implementation (Less investment in PHC – 30% of the insufficient budgets to facilities coupled with long distances to health facilities, Less investment in NCD prevention ….)  2) 1) Insufficient budget allocation for medicines, given the growing population | N/A | N/A |
| **Equity** | | | | |
|  | Facilitators | 1) Formulate to allocate resources in districts to reduce equity aspects in the allocation  2) Health policy design targets everyone | N/A | N/A |
|  | Barriers | N/A | N/A | N/A |
